# Cross-sectional imaging in cancers of the head and neck: how we review and report

**DOI:** 10.1186/s40644-016-0075-3

**Published:** 2016-08-03

**Authors:** Dechen Wangmo Tshering Vogel, Harriet C. Thoeny

**Affiliations:** Department of Diagnostic, Interventional and Pediatric Radiology, Inselspital, Bern University Hospital, Berne, Switzerland

## Abstract

Cancer of the head and neck is the sixth most frequent cancer worldwide and associated with significant morbidity. The head and neck area is complex and divided into various anatomical and functional subunits. Imaging is performed by cross-sectional modalities like computed tomography, magnetic resonance imaging, ultrasound and positron emission tomography-computed tomography, usually with fluorine-18-deoxy-D-glucose. Therefore, knowledge of the cross-sectional anatomy is very important. This article seeks to give an overview of the various cross-sectional imaging modalities used in the evaluation of head and neck cancers. It briefly describes the anatomy of the extracranial head and neck and the role of imaging as well as the imaging appearance of tumours and their extension to lymph nodes, bone and surrounding tissue. The advantages and disadvantages as well as basic requirements of the various modalities are described along with ways of optimizing imaging quality. A general guideline for prescription of the various modalities is given. Pitfalls are many and varied and can be due to anatomical variation, due to pathology which can be misinterpreted and technical due to peculiarities of the various imaging modalities. Knowledge of these pitfalls can help to avoid misinterpretation. The important points to be mentioned while reporting are also enumerated.

## Background

### Epidemiology

Cancer of the head and neck, excluding cancer of the skin and lymphoma is the sixth most frequent cancer worldwide with a current estimation of incidence being about 600,000 per year and deaths resulting in about 300,000 per year [1]. The reported incidence rate in 2008 was 6.8 % [2]. There is a geographical variation in the distribution of different head and neck cancers due to differences in genetic susceptibility, cultural risk factors like smoking, drinking, betel nut chewing, prevalence of nutritional deficiencies, difference in diet, socioeconomic status and presence of infectious agents, particularly the human papillomavirus (HPV particularly type 16), the Epstein Barr virus and human immunodeficiency virus (HIV). Certain tumours also show a gender difference with squamous cell cancers being more common in males and thyroid tumours more common in females [1–3]. Radiation exposure is an established risk for development of thyroid cancer and for mucoepidermoid carcinoma of the salivary glands [2, 4].

There have been reported associations with environmental toxins like formaldehyde [5].

Patients with head and neck squamous cell cancer can have synchronous and metachronous primary cancer of the upper aerodigestive (AE) tract. They are also at risk of developing lung cancer, oesophageal and gastric cancer usually due to combined smoking and alcohol consumption. The annual incidence of second primary cancer following successful therapy of a tumour has been reported as 3-7 % [2].

## Anatomy

The anatomy of the head and neck is very complex and knowledge of anatomy is imperative for head and neck radiologists in order to accurately localise the disease process and its relationship to adjacent structures and to make the correct diagnosis. On cross-sectional imaging, the extracranial head and neck can be divided into various anatomical spaces by fascial planes, each space having different contents and different pathologies.

**Fig. 1:**
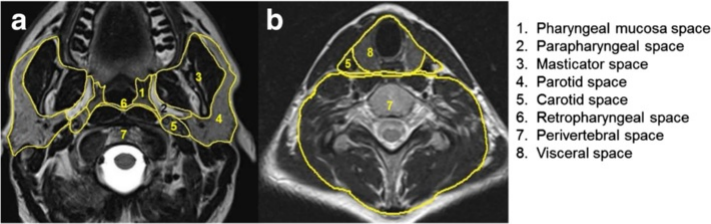
Axial T2-weighted images showing the deep spaces of the suprahyoid neck at the level of the oropharynx **a** and the infrahyoid neck at the level of the thyroid **b** which are demarcated by the deep fascia of the neck. The retropharyngeal space is a potential space between the perivertebral space and the pharyngeal mucosa space above and the visceral space below the level of the hyoid bone

The suprahyoid neck is divided into the pharyngeal mucosa space, the masticator space, the parapharyngeal space, the parotid space, the carotid space, the retropharyngeal space and the perivertebral space including the vertebrae (Fig. 1a).

The infrahyoid neck spaces consist of the visceral space including the trachea, esophagus, thyroid and parathyroid glands, the carotid space, the retropharyngeal space and the perivertebral space which extend downwards from the suprahyoid neck (Fig. 1b).

The carotid, perivertebral and retropharyngeal spaces are long vertical spaces extending from the skull base into the chest. The retropharyngeal space is a potential space bounded anteriorly by the visceral fascia and posteriorly by the prevertebral fascia and extends from the base of the skull to the posterior mediastinum till about the level of the carina. The perivertebral space includes the longus colli muscles, the paraspinal muscles, vertebrae, vertebral artery and vein and the spinal cord.

Knowledge of these spaces and their displacement patterns help in localisation of the lesions to the various spaces and facilitates the correct diagnosis. Table 1 contains a list of the various spaces with their contents and the tumours which can arise in each space. These spaces also act as potential pathways for spread of disease. Malignant tumours can grow across spaces and are transpatial or tumours can be multispatial in different spaces simultaneously, for example lymphoma and metastases.

**Table 1:** Deep spaces of the neck with contents and tumours which can arise

	Space	Contents	Tumours
1	Pharyngeal mucosa space	Mucosa with minor salivary glands Lymphoid tissue Pharyngobasilar fascia Levator and constrictor muscles Cartilaginous eustachian tube	Squamous cell carcinoma of pharynx Minor salivary gland tumours, lymphoma
2	Parapharyngeal space	Fat Minor salivary glands Trigeminal nerve (V3) Lymph nodes Internal maxillary / ascending pharyngeal artery	Lipomas, liposarcoma Minor salivary gland tumours Schwannomas Soft tissue sarcoma Metastatic lymph nodes
3	Masticator space	Muscles of mastication Mandibular ramus and body Inferior alveolar nerve	Sarcoma of muscle or bone, metastases Lymphomas, direct spread of carcinoma, Minor salivary tumour,
4	Parotid space	Parotid gland Intraparotid lymph nodes Facial nerve (VII) External carotid artery / retromandibular vein	Salivary gland tumours like mucoepidermoid carcinoma, adenoid cystic cancer etc. Metastatic adenopathy Lymphomas Oncocytomas, schwannomas, lipomas
5	Carotid space	Cranial nerves IX-XII Sympathetic nerves Jugular chain nodes Carotid artery / jugular vein	Schwannomas Neurofibromas Paragangliomas Metastatic adenopathy
6	Retropharyngeal space	Lymph nodes Fat Ectopic salivary gland tissue	Metastatic lymphadenopathy, lymphomas Salivary gland tumours Rhabdomyosarcomas
7	Perivertebral, vertebral	Vertebrae Prevertebrale muscles Blood vessels Paraspinal muscles Phrenic nerve	Primary and secondary bone tumours Plasmacytoma Sarcomas, chordoma Lymphomas Metastatic lymph nodes
8	Visceral space (infrahyoid neck)	Oesophagus Thyroid glands Parathyroid glands Trachea Nerves and vessels	Squamous cell carcinomas Minor salivary gland tumours Sarcomas Thyroid gland tumours Parathyroid tumours Metastases

**Fig. 2:**
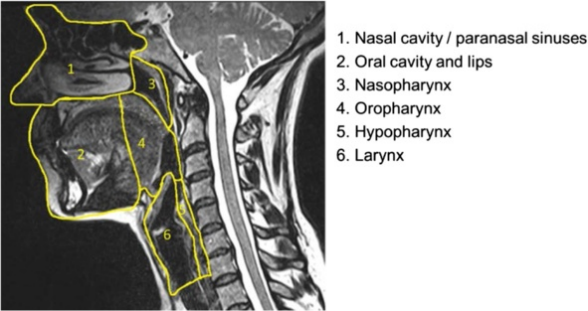
Sagittal T2-weighted image of the extracranial head and neck showing the division of the anatomical and functional units of the upper aerodigestive tract

Furthermore, the head and neck area is divided into several anatomical and functional units, with each having a different pattern of tumour spread and T staging (Fig. 2).

These include the:

1. The nasal cavity and paranasal sinuses (PNS)
2. The oral cavity and lips, the buccal mucosa, the upper and lower alveolar ridges, retromolar gingiva, floor of the mouth, hard palate and the tongue anterior to the circumvallate papillae
3. The nasopharynx
4. The oropharynx consisting of the soft palate including uvula, tonsils, base of the tongue and pharyngeal walls
5. The hypopharynx or laryngopharynx consisting of the pyriform sinus, postcricoid area and posterior hypopharyngeal wall
6. The larynx which is divided into the supraglottic portion (suprahyoid epiglottis with both lingual and laryngeal surfaces, aryepiglottic folds, arytenoid cartilages, the false vocal cords and the ventricles), the glottis (true vocal cords and the anterior and posterior commissures) and the subglottis which starts below the true vocal cords to the inferior margin of the cricoid
7. The major and minor salivary glands
8. The thyroid gland

## Tumours

Tumours can be benign or malignant and can arise from the skin, the mucosa, the major and minor salivary glands, the thyroid, the lymph nodes, the mesenchymal tissues including the bone, cartilage, fibrous tissue, fat, muscles and nerves. They can arise locally or be part of a systemic malignancy. 90 % of head and neck neoplasms consist of epithelial malignancies of the mucosal membranes of the upper AE tract called head and neck squamous cell cancer (HNSCC). The second group consists of glandular tumours arising in the major and minor salivary glands and in the thyroid gland. Skin cancer and non-melanoma skin cancer of the head and neck are considered a separate group. Other less frequent tumours include localised lymphoma, sarcomas of bone and soft tissues, and neuroectodermal tumours like paraganglioma, olfactory neuroblastoma, neuroendocrine carcinoma and malignant melanoma [2]. Very superficial lesions of the mucosa are easily seen clinically, but may not always be identified on cross-sectional imaging due to their small volume.

Treatment options include surgery, chemotherapy and radiotherapy. Treatment varies according to the type of tumours and their site of origin. We mainly need to detect and localise the tumour and define the extent of spread, the final diagnosis is then made by the pathologist.

## Imaging modalities

The cross-sectional imaging modalities available include computed tomography (CT), magnetic resonance imaging (MRI), ultrasound (US) and positron emission tomography-computed tomography with fluorine-18-deoxy-D-glucose (^18^FDG PET-CT). Each modality has its own strengths and weaknesses; these are enumerated below.

## Ultrasound

This is easily available, entails no radiation, is portable, cheap, has a high resolution and assessment of blood vessels and tumour vascularity can be performed in real time without giving contrast media. Intravascular contrast media (SonoVue^R^) can be given to evaluate enhancement of lesions. Ultrasound is ideal for guiding interventions where the lesion is well visualised.

The disadvantages of ultrasound include operator dependency and the fact that sound waves are not transmitted through bone and air, resulting in nonvisualisation of structures behind bones and air collections. Very large lesions can be difficult to evaluate completely and multiple lesions require conscientious documentation.

## Computerised tomography (CT)

This is usually the first modality used because it is widely available, relatively cheap, quick and easy to perform and reproducible. The examination time is short with less motion artefacts than an MR scan. Thin slice, high resolution image acquisition allows high quality multiplanar reconstructions with superior evaluation of bony structures and calcifications. The study can be easily extended to the rest of the body for staging purposes. Evaluation of CT images is also easier than evaluation of MR images.

Disadvantages of CT include radiation exposure, poorer soft tissue contrast compared to MRI, the need to inject iodinated contrast medium to improve contrast with the risk of contrast induced nephropathy and artefacts due to dental amalgam or orthopaedic material, if present. Contraindications to CT can be absolute or relative and include allergy to contrast media, kidney failure with some remaining function, hyperthyroidism, thyroid cancer, bodyweight above 200 kg and inability to lie down. Children are preferentially scanned with MRI or ultrasound when feasible to avoid radiation exposure.

## Magnetic resonance imaging (MRI)

This has many advantages: good soft tissue contrast, multiplanar scanning, evaluation of blood vessels without giving contrast media, no radiation exposure, no iodine containing contrast needs to be given. Functional imaging like diffusion-weighted imaging, perfusion and dynamic enhancement studies can be performed.

The main disadvantages include higher costs and longer acquisition time. The investigation is more complex with many possible sequences which can be performed and evaluation of the images is also more demanding. There are a multitude of potential artefacts (motion artefacts, flow artefacts, field distortion artefacts due to metal or at air bone interfaces or with blood products) which can mimic or obscure pathology. The face, neck and base of the skull have multiple soft tissue, air and bone interfaces with increased chance of susceptibility artefacts. Additionally movement and swallowing artefacts can degrade image quality. Examination of larger body areas is limited by longer duration of image acquisition. Gadolinium containing contrast agents should be given to evaluate tumours and abscesses. Contraindications to the use of gadolinium are absolute or relative and include previous or pre-existing nephrogenic systemic fibrosis (NSF), previous anaphylactic reaction to gadolinium containing contrast agents, patients with a glomerular filtration rate below 30 ml/min/1,73 m^2^, unstable renal impairment, hepatorenal syndrome or chronic liver function disorders and pregnancy. The risk-benefit of giving Gadolinium has to be discussed with the referring physician. Since macrocyclic compounds of gadolinium are known to be more stable than linear compounds of gadolinium, they should be preferably given if absolutely necessary.

There are also absolute and relative contraindications to performing MRI. A body weight over 200 kg precludes an MRI as the table cannot carry this weight. Other contraindications include ferromagnetic metal fragments near vital structures, metal implants in the area of interest, older pacemakers, nerve stimulators, pumps, cochlear implants and stapes prostheses, claustrophobia, very ill patients, patients who cannot lie down for long periods of time due to pain or increased risks of aspiration.

## Positron emission tomography-computed tomography with fluorine-18-Deoxy-D-Glucose (^18^FDG PET-CT)

The advantages include evaluation of the whole body including the area of interest, lymph node involvement, detection and exclusion of distant metastases and the high sensitivity of detection of glucose uptake. One big disadvantage is that it is nonspecific as uptake is also increased in inflammation. It is also more expensive, time consuming, requires patient to be fasting for 6 h, the scanning time is 20–30 min and the total investigation time is about 2–3 h. There is some radiation exposure which varies depending on whether a low dose CT is done for attenuation correction or a diagnostic quality CT is performed. Interpretation can be difficult in areas of complex anatomy, physiological variants and unusual patterns of high FDG- uptake in the head and neck.

PET image evaluation is mainly performed by qualitative visual assessment. Visual assessment requires definition of a threshold for judgement of existence and degree of radiotracer concentration. A semi-quantitative parameter currently used for PET is the standardised uptake value, which is defined as the tissue concentration of tracer within a lesion divided by tissue density, as measured by PET, divided by the injected dose normalised to patient weight multiplied by a decay factor. Instead of body weight, the injected dose may also be corrected by the lean body mass, or body surface area (BSA). Different tumours also show different glucose uptake and there is no standard threshold value for diagnosis of tumours, however it can be useful for therapy monitoring.

## Role of imaging

Clinical examination and endoscopy can visualise tumours in the mucosa of the upper AE tract. However, not all areas are well visualised and submucosal and deeper tumours cannot be seen.

The role of imaging lies in the detection or exclusion of tumours, the characterisation of tumours if present and also to delineate it’s size and extent, to see which structures are involved, whether there is spread to regional lymph nodes, perineural or perivascular spread, bony invasion or in staging examinations, to detect metastases.

Imaging is also used for obtaining tissue diagnosis by image guided biopsies, for planning therapy (radiotherapy, computer assisted surgery), to follow patients under therapy if necessary, to evaluate response to treatment allowing change in therapy when possible and after treatment, to detect recurrences before it becomes clinically evident to improve chances of successful salvage.

## Primary imaging (before therapy)

CT and MRI are commonly used and provide most of the relevant information when the study parameters are optimised. Information provided by the different modalities are usually complementary. The choice of the primary imaging modality depends on what the clinician wants to know, the available technology, cost factors, reimbursement policies, clinician acceptance and patient factors like presence of contraindications to MRI (older pacemakers, metal fragments, aneurysmal clips) or contraindications to the use of iodinated contrast media (allergy, renal failure, thyroid cancer). For paediatric patients, radiation exposure is an important factor to consider and therefore MRI should be performed in children-if available.

The ideal initial study is one which is safe and provides all the information required for proper management. This depends on the site of origin of the tumour and known patterns of spread. The next study, if required should be able to answer questions not clarified by the first study.

Furthermore, distant metastases or concomitant tumours which can be present due to common risk factors should be excluded. This is usually done either by performing a whole body CT or PET-CT. There are increasing reports of PET-MRI, but the availability is limited.

Once the presence of a tumour is established, one has to correctly localise the tumour and identify its extent.

## Appearance of tumour and tumour extension

**Fig. 3:**
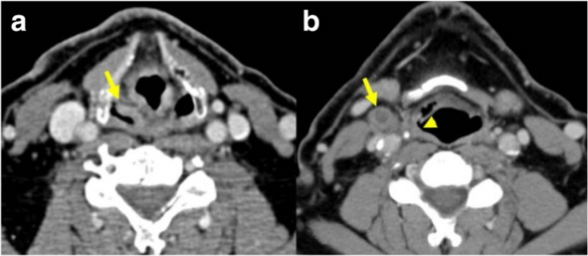
Patient with cancer of the right pyriform sinus. Contrast–enhanced CT scan **a** showing asymmetrical thickening of the pyriform sinus (arrow) on the right. CT scan at a lower level **b** showing slight thickening of the posterolateral hypopharyngeal wall (arrowhead) and an adjacent normal sized lymph node with central necrosis (arrow) indicating metastasis

**Fig. 4:**
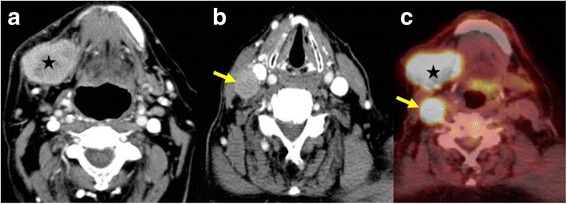
Patient with an undifferentiated cancer of the right submandibular gland. Contrast–enhanced CT scan **a** showing the solid, heterogeneously enhancing mass on the right side (star). CT scan at a slightly lower level **b** shows the solid metastatic lymph node (arrow). PET-CT image **c** showing the intense uptake of FDG glucose by the tumour (star) and the lymph node metastasis (arrow), both seen at the same level due to difference in the degree of neck flexion

**Fig. 5:**
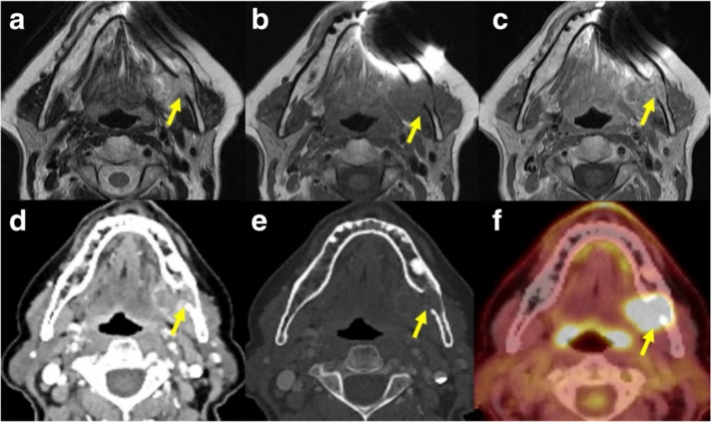
Patient with carcinoma ex-pleomorphic adenoma of the submandibular gland on the left with invasion of the mandible. In the T2-weighted MR image **a** the lesion is heterogenously hyperintense to muscle and there is loss of the cortical hypointense line indicating bone invasion (arrow). In the T1-weighted image **b** the lesion is hypointense to the muscle and there is replacement of the normal high signal marrow fat by the tumour. The T1-weighted image **c** after injection of gadolinium without fat saturation shows some enhancement of the lesion. Contrast enhanced CT scan at the same level **d** shows the heterogenously enhancing mass which extends into the mandible. The cortical bone erosion (arrow) is seen better in the bone window setting **e**. The PET-CT scan **f** at the same level showing the increased glucose uptake by the tumour with extension into the mandible

Tumours appear as asymmetrical thickening of the mucosa (Fig. 3a) or a mass of soft tissue density on CT which enhances according to its vascularity (Fig. 4a). Larger tumours can show areas of necrosis. On MRI, (Fig. 5a-c) the tumour is usually hyperintense to muscle on T2-weighting and hypointense or isointense to muscle on T1-weighting. The precontrast T1-weighted images are particularly useful in differentiating tumour from surrounding fat, detecting neurovascular bundle encasement and bone marrow involvement. MRI can overestimate tumour if inflammatory changes and bleeding is present. The use of diffusion-weighted magnetic resonance imaging (DW-MRI) can help to better detect and demonstrate tumour since most squamous cell cancers are relatively hypercellular and can show diffusion impediment. This is especially true and useful in patients who are imaged after surgery, where there is surgical change in the anatomy and edema and inflammation due to therapy.

On PET-CT, the morphological changes due to the tumour are seen as in CT with increased uptake of glucose in those tumours which are FDG avid (Figs. 4c, 5f), other processes resulting in glucose uptake like inflammation or biopsy will however also show increased uptake and should be excluded when possible.

Bony erosion appears as an interruption of the hypointense cortical rim on MR (Fig. 5a-c) or the peripheral, cortical hyperattenuating rim on CT (Fig. 5d,e). Detection of subtle erosions require thin section CT with bone algorithm reconstruction in multiple planes. Sclerosis can be a sign of tumour infiltration or reactive osteitis. Medullary involvement is better detected with MRI. The replacement of normal hyperintense fat signal in the bone marrow on native T1-weighting with enhancing tissue indicate marrow involvement. Unfortunately, these changes can also be seen in peritumoral edema, inflammation, coexisting periodontal diseases, osteomyelitis, radiation fibrosis and osteoradionecrosis.

**Fig. 6:**
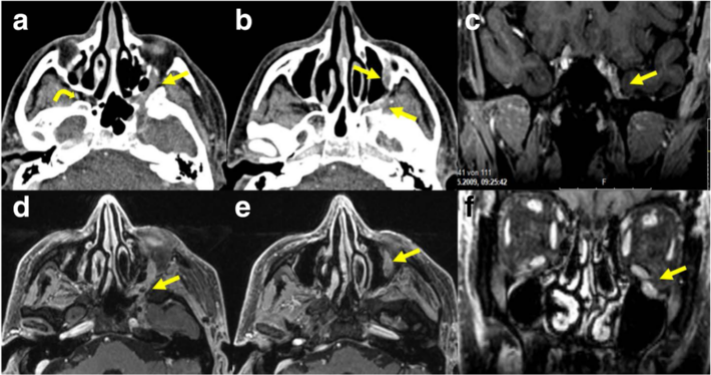
Patient with adenoid cystic carcinoma and perineural extension along the inferior orbital nerve, the pteryopalatine fossa and the maxillary nerve on the left side. Contrast enhanced CT images **a**, **b** showing the asymmetric thickened enhancing nerve (arrows) on the left. The curved arrow **a** shows the normal fat density of the pterygopalatine fossa on the right side. Axial T1-weighted images **d**, **e** showing the thickened enhancing nerve (arrows) on the left. Coronal T1-weighted images with fat saturation after injection of gadolinium **c** showing asymmetrical nerve enhancement (arrow) in the cavernous sinus and **f** the thickened infraorbital nerve on the left (arrow)

Perineural spread on CT is diagnosed when there is enlargement or destruction of the respective nerve foramina with replacement of normal fat signal in the soft tissues (Fig. 6a,b). On MRI, the nerve may be thickened and/or show increased enhancement (Fig. 6a-f). Perineural spread can be antegrade or retrograde and skip lesions can occur. This finding can also be seen in inflammatory changes or due to edema of the nerves. Secondary signs of denervation such as atrophy of the muscles innervated by the involved nerve, or abnormal enhancement of the involved muscles or clinical signs of inflammation can be helpful in this respect.

PET-CT signs of nerve involvement include linear thickening or linear increased/decreased FDG activity along the expected course of the cranial nerve. Abnormal activity can be seen in the muscles supplied by the nerve or increased FDG activity in the synergistic/antagnostic muscles which overcompensate to maintain function where possible. Other signs like atrophy and foramen changes are seen on the CT images [6].

Metastases to adjacent Iymph nodes is an important prognostic factor. We use an image based classification of cervical Iymph nodes described by Som et al. [7], where lymph nodes are divided into seven stations in the neck according to their position. The retropharyngeal, occipital, facial, parotid and mastoid lymph nodes are not included in this classification and when involved are named according to their anatomical site. Most metastatic nodes are enlarged, a transversal short axis diameter of more than 1.5 cm for Level II nodes, and more than 1 cm for all other nodes in the neck are considered pathological (Fig. 4b). However, normal sized nodes can have focal areas of necrosis indicating metastases (Fig. 3b). This should be differentiated from the fatty hilus by carefully looking at the native scans and measuring the Hounsfield units, necrosis also tends to be central while the hilus is eccentric [8]. Other features include increased enhancement, irregularity in shape, heterogeneous enhancement and stranding or obvious infiltration of the adjacent soft tissue which imply extracapsular spread. Extracapsular spread is an important finding to be mentioned in the report since it is a marker of increased locoregional failure if only surgery is performed and chemoradiation should also be given.

**Fig. 7:**
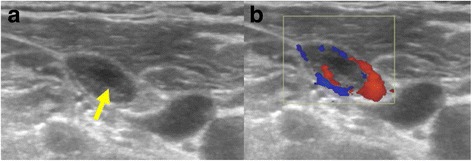
Lymph node metastasis **a** in a patient with known multifocal papillary carcinoma of the thyroid showing a small area of necrosis (arrow). Color doppler image showing pathological peripheral vascularity **b**

Ultrasound can evaluate the morphology of lymph nodes and Doppler sonography can be used to assess the vascular pattern. Monitoring the lymph node size is also useful in the assessment of treatment response. Enlargement of lymph nodes indicates pathology but since reactive lymph nodes also enlarge, it is nonspecific. A round shape with a short to long axes ratio greater than 0.5 is a useful pointer, except for submandibular and parotid nodes which can normally be round. Eccentric cortical hypertrophy can indicate focal tumor infiltration. The lymph node border may be smooth or irregular, irregular borders in confirmed metastases indicate extracapsular spread. Loss of the echogenic hilus, necrosis (Fig. 7a) and fine calcification can all be seen in metastases. Normal lymph nodes have hilar vascularity and the presence of peripheral vascularity in lymph nodes is a useful indicator of malignancy [9] (Fig. 7b). On PET-CT metastatic lymph nodes show increased uptake (Fig. 4c).

## Tumour staging

Tumours are classified into stages which determine the appropriate treatment and prognosis. The union for international cancer control (UICC) has published a staging system UICC TNM classification that is internationally accepted. It is based on the primary tumour site characteristics like size and spread (T), involvement of Iymph nodes (N) and the presence or absence of metastases (M). The use of this standard staging helps in treatment planning, in follow up and allows exchange of information between treatment centres. The anatomically based TNM classification considers the local, regional and distant extension of tumours. Due to the numerous subsites in the head and neck, each with different T staging, readers are referred to the UICC TNM classification of malignant tumours [10].

N staging of lymph nodes is based on the site, number and laterality of the lymph nodes relative to the primary tumour site. It does not take into account the presence of extracapsular spread of tumours or involvement of retropharyngeal lymph nodes.

- *N staging for most tumours (except the nasopharynx and thyroid)*
- Nx means that the regional lymph nodes cannot be assessed
- N0 no node involvement
- N1 single ipsilateral lymph node 3 cm or less in greatest diameter
- N2a single ipsilateral lymph node between 3 and 6 cm
- N2b multiple ipsilateral lymph nodes less than 6 cm
- N2c contralateral or bilateral lymph nodes less than 6 cm
- N3 any lymph node larger than 6 cm.
- *N staging of nasopharyngeal tumours (midline nodes are considered ipsilateral).*
- N0 no node involvement
- N1 ipsilateral single lymph node above the supraclavicular fossa, and/or unilateral or bilateral retropharyngeal lymph node(s), 3 cm or less in greatest dimension
- N2a ipsilateral single lymph node between 3 to 6 cm
- N2b multiple ipsilateral lymph nodes less than 6 cm
- N2c contralateral or bilateral lymph node(s) 6 cm in greatest diameter above the supraclavicular fossa
- N3a lymph node or nodes larger than 6 cm, unilateral or bilateral
- N3b extension to the supraclavicular fossa
- *N staging for thyroid cancer*
- N1a level VI (pretracheal, paratracheal, prelaryngeal /Delphian lymph nodes)
- N1b unilateral, bilateral or contralateral cervical (level I-V), retropharyngeal or superior mediastinal (level VII) nodes.

The presence of metastases is another important prognostic factor. M0 means there is no distant metastasis and M1 indicates spread to distant organs beyond the regional nodes.

Therapy depends on the tumour type and staging and consists of surgery and/or chemotherapy and/or radiotherapy. Therefore, the correct diagnosis and adequate assessment of tumour extent is essential for proper therapy of the tumours.

## Imaging appearance after therapy

**Fig. 8:**
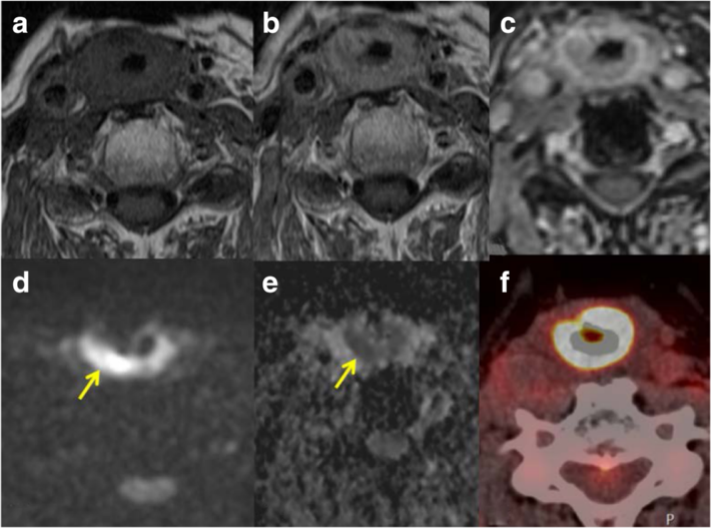
Axial T1-weighted MR image before contrast **a** T1-weighted MR image after injection of gadolinium without fat saturation **b** and with fat saturation **c** in a patient with diffuse thickening of the neopharynx (radial free flap reconstruction) after total laryngectomy for larynx cancer. DW-MRI shows hyperintense signal (arrow) in the b800 image **d** with corresponding low signal in the ADC map **e** indicating tumour recurrence. The PET-CT image **f** showed intense uptake of glucose (see also Fig. 10) over a long segment due to which it was interpreted as inflammation

Imaging appearance after therapy depends on the type of treatment received and this should be known before evaluation of images. The expected changes after radiotherapy include edema and inflammation with thickening, swelling and induration of all the structures in the radiation field, maximal changes are seen during the first few months after the end of radiotherapy. These changes decrease with time and can be followed by atrophy and shrinkage as in postradiation sialadenitis. Calcification may also occur especially in lymph nodes, dystrophic calcification after therapy is a potential cause of false positive FDG PET. The changes after surgery depend on the surgical procedure which has been performed. Surgery may be extensive with removal of significant tissue which requires reconstruction with pedicled or free soft tissue flaps, grafts and prosthesis. Neck dissection performed to remove lymph nodes can be selective or radical. Accordingly there can be significant changes in the anatomy and it can be difficult to detect tumour in this background of altered anatomy. Tumour recurrences appear as a soft tissue mass at the primary site on CT or MRI and after major surgery with reconstruction, recurrence is usually seen at the edge of the resection or the soft tissue flaps. It can be very difficult to distinguish between tumour recurrence and posttherapeutic changes in the early stages [11]. A baseline scan performed about 3–6 months after the end of therapy can be very helpful in this respect. PET-CT has a high negative predictive value but false positive results occur due to the inflammatory post therapeutic changes. DW-MRI is useful in detecting tumour in the background of posttherapeutic inflammation [12, 13] (Fig. 8a-e).

Patients with complications after treatment may present for imaging. Early surgical complications like fistula formation can be diagnosed on fluoroscopy or on CT after giving oral contrast media. Late complications of radiotherapy like osteonecrosis and soft tissue necrosis can be seen well on both CT and MRI scans although it can sometimes be difficult to differentiate from tumour recurrences or infections. Radionecrosis occurs after radiotherapy, the presence of air, sclerosis and fragmentation of the bone points to radionecrosis.

## Prescribing

The modality prescribed depends primarily on availability. In developed countries most modalities are available and physician’s preference, experience, expertise, the area of interest and the information required determines what is prescribed. As a general guide, MRI is best for evaluation of the oral cavity and oropharynx and for evaluation of perineural tumour spread. For evaluation of the PNS, nasal cavity and larynx, where soft tissue and bony details are important, both CT and MRI are commonly used and provide complementary information. The thyroid gland is well evaluated by ultrasound, MRI or CT, the use of iodinated contrast media is contraindicated in cases of suspected thyroid cancer due to therapeutic implications. The salivary glands can be evaluated by all cross-sectional modalities. For evaluation of the lymph node status, all modalities can be used. MRI is preferred for evaluation of perineural spread.

Ultrasound is used widely for evaluation of the thyroid gland, neck lymph nodes and salivary glands and in soft tissue mass evaluation and in the paediatric population. Ultrasound guided fine needle aspiration cytology or biopsies can be highly accurate for neck node staging but is highly operator dependent. Also not all areas of the neck can be adequately visualised by ultrasound (e.g., deep lobe of the parotid, retropharangeal lymph nodes) and a CT or MRI may be required for completion of staging.

PET-CT is useful for detection of the primary tumour, known or unknown, nodal metastases, second tumours or distant metastases. It has a role in restaging and long term surveillance to detect recurrence.

In our institute, patients with clinically known tumours undergo a CT scan of the neck as the primary investigation. A complementary MRI is done when possible for better tumour delineation in patients with tumours of the oropharynx, oral cavity, nasopharynx, PNS and for exclusion of perineural tumour spread. Conventional CT of the thorax and abdomen is done for staging of early tumours T1-2 N0. PET-CT is performed for advanced tumours T3-4 where there is increased risk of metastases, in all patients with lymph node metastases and for cancer of unknown primary (CUP). Most patients also undergo panendoscopy and all patients are biopsied. Ultrasound is usually reserved for evaluation of the thyroid glands and for guiding biopsies in adults. Children presenting with a swelling are preferentially evaluated first with ultrasound and then as required, with MRI, in order to avoid radiation exposure.

After completion of therapy, patients are assessed clinically and radiologically to detect tumour persistence / recurrence or metastases. The radiological follow up after therapy is individualised and depends on tumour type and tumour stage, but the general scheme applied in our institute is a CT or MRI scan 3–4 months after completion of therapy, which is repeated after about 1 year and 2 years. Patients with stage III and IV cancers undergo restaging with PET-CT at 4–5 months after end of therapy.

## Image display

**Fig. 9:**
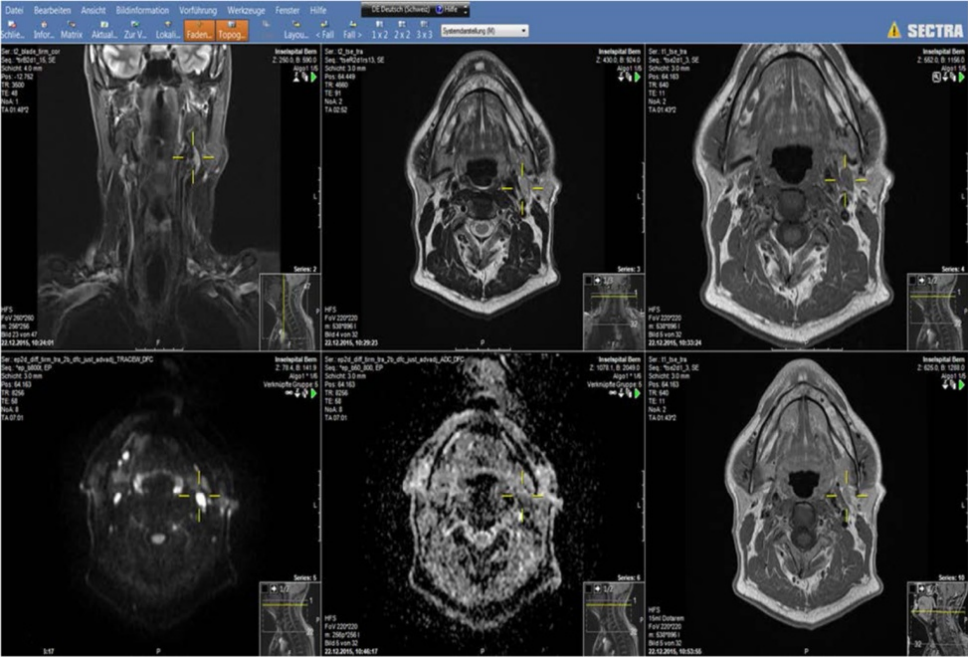
PACS system Sectra Workstation IDS7 Version 17.1.10 image display. Many images can be evaluated simultaneously. The localiser function helps to quickly localise findings in different planes and sequences

We use a PACS system Sectra Workstation IDS7 Version 17.1.10. Ideally two 21 inch or one 30 inch standard radiology monitor for image display with additional monitors for reporting should be available. It should be possible to view multiple images / series simultaneously. The different sequences can be tagged to move together thus improving efficiency and accuracy. This is especially important for the DW-MR images where the spatial resolution may be suboptimal. The use of the localiser function of the PACS system allows rapid localisation of findings in different planes. Different modalities can be viewed simultaneuously. There should be a capability for multiplanar reconstructions so that individualised reconstructions can be made as required. Multiple image viewing is especially important for comparisons of scans during follow up (Fig. 9).

**Fig. 10:**
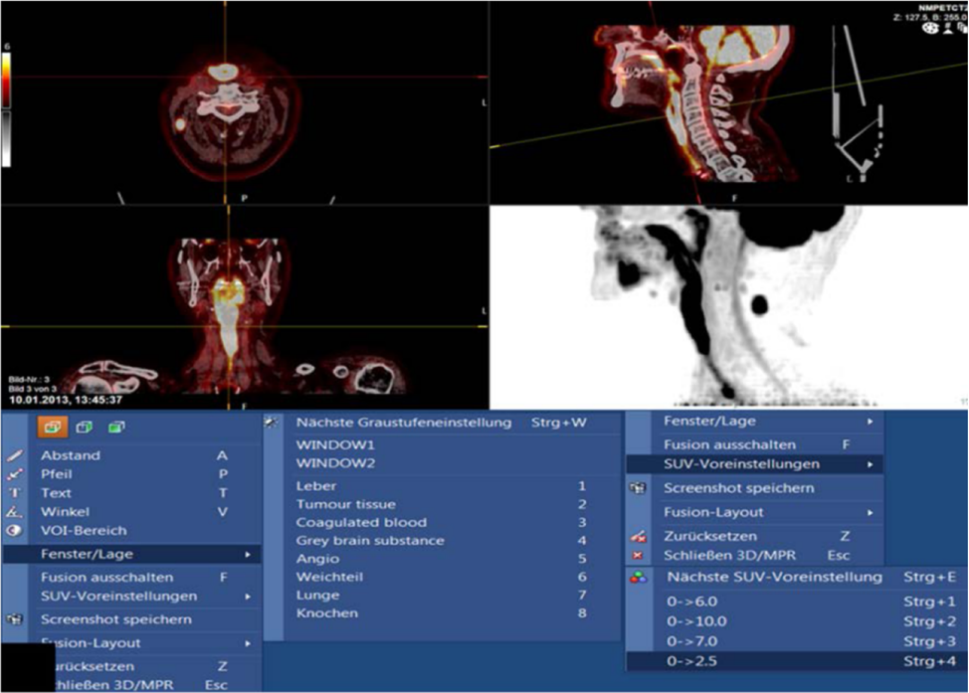
PACS system Sectra Workstation IDS7 Version 17.1.10 with NM Fusion image display allowing demonstration of the volumetric PET-CT data in all three planes and as MIP. The various preset window and SUV settings are shown in the bottom half. The PET images shows the long segment of avid enhancement of the neopharynx in a patient with tumour recurrence (same patient as in Fig. 8) and a lymph node metastasis on the right

For PET-CT images, the three dimensional volume data is depicted in the coronal, sagittal and axial planes with maximum intensity projections (MIP). The NM Fusion software in the PACS allows fusion of the PET and CT images and enables the three dimensional image display. There are different preset window settings and SUV settings which can be selected (Fig. 10).

## Basic requirements and image optimisation

### Ultrasound

The lesion should be accessible to ultrasound without any intervening bone or air.

Proper patient positioning with good access to the structure to be evaluated is important. One should use adequate gel and the correct frequency of the transducer. The higher the frequency of the transducer the better the resolution but the penetration into the deeper tissues is restricted and for scanning deeper tissues, the frequency has to be reduced. We use multifrequency transducers where the frequency is adjusted during the scanning. It is important to know the limitations of ultrasound and to advise either a CT or MRI when required.

## CT

State of the art CT requires multidetector CT. Volumetric data are acquired rapidly, reducing volume averaging artefacts and motion artefacts and allowing high quality retrospective reformations. One disadvantage is the higher radiation dose. Since CT has poor soft tissue contrast, intravenous iodine containing contrast agents have to be injected. Delay between injection and scanning is essential to allow time for the contrast to reach the area of interest.

Proper positioning of patient supine with neck in slight extension is important. The head should be positioned as symmetrically as possible, the patient should be as comfortable as possible to avoid movement. Dental amalgam can seriously degrade image quality and in patients with tumours of the oral cavity, scanning can be done with a tilted CT gantry or with an open mouth. The puffed air technique which entails puffing the cheeks out with air during scanning improves delineation of oral mucosal lesions and allows identification of site of origin of tumours. Using a modified Valsalva maneuver, which consists of expiration against the resistance of pursed lips or a pursed nose and holding the breath for at least 10 s during scanning, dilatation of the hypopharynx and its visualisation can be improved. E phonation, which is performed by having the patient say ‘e’ uniformly for at least 10 s during scanning, also results in better depiction of the true and false vocal cords and the laryngeal ventricles with better localisation of site of origin of tumours.

Scanning factors can be optimised. The field of view (FOV) should be as small as possible to optimise spatial resolution. Image reconstructions are done using soft tissue windows and bone windows. Lung windows should be used to evaluate the apical lungs which are also included in the lower neck scans to exclude metastases. Volumetric scanning allows reconstruction of the images in the correct plane, for example for larynx cancers along the plane of the vocal cord. Three dimensional reformatting is usually used for evaluation of bony structures. 3D volume rendering and virtual endoscopy can also be performed.

From skull base to the oral cavity, image plane should be parallel to the hard palate, from the oral cavity to the thoracic inlet, image plane should be parallel to the vocal cords which is practically parallel to the intervertebral disc space at C 4–5 and C 5–6 level.

## MRI

A 1.5 or 3 Tesla MRI scanner is required. For lesions near the skull base, a head coil is used and for lesions lower in the neck, a neck coil can be used. The use of a single receiver prevents evaluation of the Iymph nodes status. The combination of a head and a neck coil permits evaluation of the whole head and neck area. Disadvantage of combination of coils is the change in the recipient field characteristics at the crossover between two coils. Meticulous shimming, coil and patient positioning as well as the use of sequences with short echo trains can help overcome this drawback [14].

The patient should be supine, the head and neck should be aligned and symmetrically positioned. The patient should be positioned comfortably with slight head fixation to reduce motion artefacts. The patient should not cough during image acquisition. Patient should be encouraged not to speak or swallow during image acquisition even though it may not always be feasible. Sedation of children can reduce movement.

Sequences: The standard sequences used in our institute include, a coronal inversion recovery sequence, axial T1-weighted and T2-weighted images over the area of interest, DW-MRI with three b values (50, 300, 800), post contrast T1-weighted axial images and a volumetric interpolated brain examination (VIBE) with fat saturation over the whole neck. For evaluation of the larynx and hypopharynx, 3 mm slice thickness is mandatory. Otherwise 3–4 mm axial slice thickness is used. The 3D VIBE is very good for evaluation of perineural spread. In the presence of metal, fat saturation should not be performed. Perfusion and dynamic scanning are not performed routinely.

Technical parameters can be optimised. The use of faster pulse sequences and parallel imaging if coils permit, echo train length for diffusion can shorten the scan time, thus improving image quality. CT is however much quicker and to be preferred in very sick and restless patients.

Injecting MR contrast is important as it increases the contrast between tumour and surrounding lesions improving tumour detection, necrosis is also seen better after contrast administration. Bone involvement detection requires fat saturation after contrast as the signal intensity after contrast becomes similar to the fat in the bone marrow.

DW-MRI should be performed routinely. Factors for optimisation of diffusion-weighted images include minimisation of echo train length for SE echoplanar sequences, the use of shim blocks, zoom echo-planar imaging, use of antisusceptibility cushion and parallel imaging [15].

## PET-CT

For evaluation of the head and neck, it is necessary to perform dedicated high resolution head and neck CT (HR HN PET-CT) with smaller FOV, longer acquisition time per slice position and thinner slice thickness. This is performed ideally with the arms lying parallel to the body and includes giving intravenous contrast. Ideally smaller pixels should be used, reducing partial volume effect which underestimates FDG activity in all lesions smaller than twice the spatial resolution. The use of the whole body PET-CT for evaluation of head and neck cancer is not adequate for smaller tumours.

Adequate patient preparation: patient fasting at least 6 h prior to investigation, adequate hydration, patient instructed not to chew or speak during the FDG uptake phase. Patient should be kept adequately warm for 30–60 min prior to scanning to avoid shivering and increased uptake by the muscles. All metal that can cause artefacts are removed. Proper patient positioning is important.

PET-CT in diabetics can be problematic due to elevated glucose levels with competitive inhibition of FDG in different tissues. Intravenous insulin before FDG injection can reduce glycaemia but it increases FDG uptake in muscles and fat. Monitoring serial blood sugar levels (BSL) after giving insulin and only giving FDG when the BSL is starting to rise or plateauing, minimises the muscular uptake due to insulin. Another option is to reschedule the investigation if BSL are more than 120 mg/dl.

## Pitfalls

Pitfalls can be due to variations in the anatomy, pathology which can be misinterpreted, technical factors/changes obscuring or mimicking disease. Incidental findings may be insignificant or significant and can alter patient management.

### Pitfalls due to normal anatomy

Hypertrophy of the lingual tonsils can look like tumour.

The arch of the thoracic duct in the left lower neck, Iying between the carotid and subclavian artery can mimic a lymph node on axial images. It is a tubular structure ending in a vein and can be identified by following it.

The accessory levator claviculae muscle, normal in lower mammals, is infrequently found in humans. It arises from the transverse process of the upper vertebrae and inserts on the lateral half of the clavicle. It can mimic nodes on axial scans.

The thyroid cartilage has a very variable pattern of ossification and areas of non-ossification can be misinterpreted as invasion of the cartilage.

### Pitfalls due to pathology

Recurrent laryngeal nerve palsy leads to asymmetry of the vocal cords which can be misinterpreted as a tumour.

**Fig. 11:**
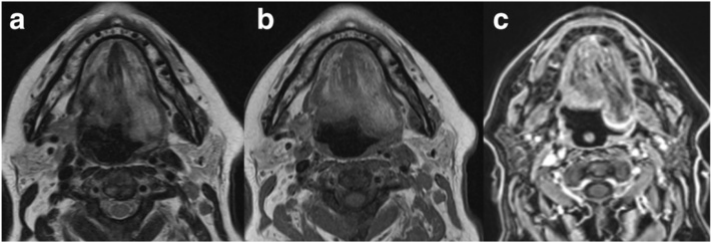
Patient with hypoglossal nerve palsy on the left. The T2-weighted MR image **a** the T1-weighted MR image **b** and the T1-weighted MRI image with fat saturation after injection of gadolinium **c** shows the fatty changes of the left side of the tongue and a dorsal bulge due to loss of muscle tone. This should not be interpreted as a tumour on the left side

Hypoglossal nerve palsy leads to fatty degeneration and loss of tone of the involved half of the tongue. There is dorsal shift of the tongue base due to gravity in the supine position leading to the appearance of a pseudotumour in the oropharynx (Fig. 11).

Inflammation of the nerves like neuritis is difficult to differentiate from perineural tumour spread, clinical correlation and serial scanning is required to resolve this.

It can be difficult to differentiate between lateral neck cysts and cystic Iymph node metastases from papillary cell cancer of the thyroid. It is also difficult to differentiate between suppurative inflammatory and necrotic metastatic Iymph nodes. The margins of the nodes can be irregular and ill-defined indicating the presence of inflammation and clinical signs of inflammation are usually present. In those cases where the clinical features are equivocal, DW-MRI might help but aspiration cytology is usually required.

Tumor recurrence and therapeutic changes can also be difficult to differentiate, DW-MRI imaging and serial scanning can help, followed by biopsy when indicated.

Bony changes due to tumour infiltration / inflammation / radionecrosis can be difficult to differentiate, here the clinical setting is important and DW-MRI might also help.

### Technical pitfalls with CT and MRI

Inadequate scan volume where the tumour may not be adequately included, inadequate contrast enhancement which might allow misinterpretation of blood vessels as Iymph nodes or too much contrast with beam hardening artefacts obscuring the tumour. Movement artefacts can obscure tumour. Pulsation artefacts and aliasing artefacts can mimic tumours. T2 shine through effect in DW-MRI can be mistaken for diffusion impediment if the ADC maps are not looked at.

### Technical pitfalls with PET-CT

There is physiological uptake by the brain, lymphoid tissue, salivary glands, muscles and brown adipose fat when present and this should not be misinterpreted as tumour. False positive readings can occur after trauma, biopsy, chemotherapy, inflammation. Increased uptake is also seen in thyroid nodules with high glucose metabolism, Warthin tumour of the parotid gland and unilateral cranial nerve palsy. Unilateral cranial nerve palsy results in compensatory hypertrophy of the muscles on the contralateral side leading to asymmetrical increased uptake of glucose on the normal side. Patients who talk or chew after FDG injection show physiological uptake in the tongue, pterygoid muscles and vocal cords.

False negative readings can occur if the patient is not fasting (inadequate preparation), diabetes with high blood sugar level or in the vicinity of structures with high glucose metabolism, due to obscuration by dental hardware, inadequate PET scanner resolution. Well differentiated, slow growing tumours or tumours with central necrosis may not show any uptake. Very small lesions with partial volume effect can be missed and in the presence of increased background noise due to volume overload or kidney failure, lesions can be missed.

Artefacts due to dental hardware and metallic implants propagate to the PET images through CT-based attenuation correction factors [16].

## Incidental findings

These can be relevant or irrelevant. Relevant findings should be mentioned.

Retropharyngeal carotid artery when present should be mentioned to avoid inadvertent biopsy. Presence or absence of bony septae around the skull base in cases of computer assisted surgery in the nasal and paranasal sinuses is very important and should be mentioned.

Laryngoceles are benign but can be due to obstruction at the ventricle due to stenosis or tumour and tumour has to be excluded.

Other incidental findings like detection of other tumours or findings of systemic or local illnesses, vascular anomalies or vessel stenosis which can influence the ability of the patient to undergo surgery or radio/chemotherapy can change patient management and should be communicated to the clinician.

Irrelevant incidental findings like atheromas can be mentioned in the findings section of the report but not in the conclusion.

## Quality control

This is important especially in referral centers where the patients are referred with completed imaging scans. The quality of the investigations vary greatly between different institutions and if inadequate, they should be repeated. One should check whether the area of interest and the tumour has been completely included in the scan. The presence of artefacts, due to motion, metal or contrast media can seriously degrade image quality. For MRI check which sequences have been performed, whether the fat saturation is homogenous. For PET-CT look for undesired nucleotide deposition in the muscles, resolution and misregistration between the CT and the PET scan.

## Reporting

It is important to identify the information that the clinician requires and this should be addressed in the conclusion. A mention of the technical parameters like the different phases of contrast enhancement in CT scans and the different MR sequences performed should be made as well as a description of the body part that has been scanned. Furthermore, the name and the dose of the administered contrast medium has to be noted. We do not use report templates or include key images in our reports. Most oncologist and ENT surgeons appreciate a summary statement stating that the findings are compatible with a certain TNM stage and it should be mentioned when possible. The TNM staging of larynx cancers includes vocal cord fixation, a clinical parameter for staging and the final staging is done in the interdisciplinary tumour boards where the images and the patients are clinically evaluated at the same time. The report should include a detailed description of the tumour, its size, location, extension over the midline to the other side, the extent of local spread to bone, adjacent soft tissues, involvement of the blood vessels, whether the vessels are surrounded, invaded or only in contact. The involvement of Iymph nodes, whether unilateral or bilateral, the number of involved nodes, the location or level of the involved lymph nodes according to Som [7] and presence of extracapsular spread should be mentioned. Perineural spread, metastases to bone, brain or presence of other tumours are important. Invasion of adjacent muscles, the prevertebral muscles and for tumours of the tongue, involvement of extrinsic muscles increases the T staging.

Adequacy of the investigation and whether complementary imaging is required should be mentioned in the initial scan.

Important points to be included in the early posttherapeutic scan are the presence or absence of residual tumour, size if present and the degree of confidence for the diagnosis. In follow up scans, the presence or absence of recurrence or complications of treatment like osteoradionecrosis, ulcer, soft tissue necrosis and XII nerve palsy should be mentioned. DW-MRI sequences can help in differentiation between tumour and posttreatment changes. If the findings are equivocal, then depending on the clinical situation, patients should undergo a follow up examination or a biopsy.

Similarly for PET-CT, one should mention whether the investigation is of adequate quality.

Describe the location, extent and intensity of the pathological FDG accumulation. Describe all findings in the accompanying CT even if there is no accompanying increased glucose uptake. Mention confounding factors like inflammatory changes, muscle activity or high glucose levels at the time of the investigation. Address the clinical question asked. Compare with old investigations when available. A definite diagnosis should be made if possible, if findings are equivocal then the probability of a diagnosis should be stated.

In cases of conflicting findings between CT, MRI and PET-CT concerning lymph node metastases or bone and cartilage invasion, the positive findings should be used for staging as false negatives can occur. For lymph nodes, the known pattern of spread can additionally be used to determine the probability of metastatic involvement. False positive results can also occur regarding bone and cartilage involvement and discussion with the clinicians is very important. Communication of indeterminate findings is especially important before surgery so that the surgeon can plan for all eventualities preoperatively. All cases of head and neck cancers are always discussed in the interdisciplinary tumour conferences with the surgeons, the oncologists and the radiation therapists and treatment options are tailored to individual patients and also depend on the patient’s age, health status and personal wishes.

## Summary

The head and neck area is complex and knowledge of anatomy is essential.

The different cross-sectional images have their strengths and weaknesses.

The diagnosis is only as good as the images available. Is the study adequate? If not repeat.

Is there a mass? Is it discrete, localised or generalised? Is it confined to an anatomical space? Proper localisation of the lesion helps in reaching the correct diagnosis. Is it multi-spatial or trans-spatial? What are the normal contents of the involved space? What are the imaging appearances and enhancement characteristics? Cystic or solid? Inflammatory or infection? Malignant?

The next step? Further imaging? Which imaging modality ? Biopsy.

There are many pitfalls and one should be aware of them.

Incidental findings although seemingly irrelevant can be a clue to an underlying tumour.
